# Enhanced interfacial activity by maximizing synergy between long-chain ionic liquid and conventional surfactant for enhanced oil recovery

**DOI:** 10.1039/d4ra02092h

**Published:** 2024-06-12

**Authors:** Simin Asadabadi, Javad Saien, Mona Kharazi

**Affiliations:** a Department of Applied Chemistry, Faculty of Chemistry and Petroleum Sciences, Bu-Ali Sina University Hamedan 6517838695 Iran +98 8131408080 s.asadabadi@basu.ac.ir si.asadabadi@gmail.com saien@basu.ac.ir jsaien@yahoo.com m.kharazi@sci.basu.ac.ir kharazi.mona@yahoo.com +98 8131408080

## Abstract

Conventional surfactants encounter limitations for application in oil reservoirs; however, combining surface-active ionic liquids (SAILs) with conventional surfactants presents an opportunity to enhance the interfacial properties of crude oil–water systems, giving also economic benefits. Accordingly, blends of a long-chain cationic imidazolium-based SAIL, namely, 1-dodecyl-3-methylimidazolium chloride, [C_12_mim][Cl], and the anionic conventional surfactant, sodium dodecyl sulfate were investigated here. Initial experiments with individual surfactants revealed efficient adsorption and consistent adsorption parameters. Subsequently, the use of mixtures showed synergistic effects for interfacial tension reduction of up to 86.0%, and critical micelle concentration reduction of 72.1% compared to the linear contribution of individual components. These improvements were observed at the optimal SAIL mole fraction of 0.3 and the mixture concentration of 0.003 mol dm^−3^, resulting in interfacial tension reduction from 29.1 to 1.6 mN m^−1^ as well as achieving a low critical micelle concentration of 2.7 × 10^−3^ mol dm^−3^ coinciding with 83.6% synergy. These findings underscore the favorable interactions between oppositely charged components in the mixtures, amplifying their activity beyond the linear contributions of the individual surfactants. Additionally, theoretical assessments using the Gibbs adsorption equation and the Rosen model provided insight into the adsorption behavior of both the individual surfactants and their mixtures, together with reasonable variations in the corresponding parameters.

## Abstract 2

Mixtures of SAIL and surfactant caused high reductions in IFT, CMC and IFT at CMC alongside high synergy at ambient temperature and low total concentration.

## Graphical abstract

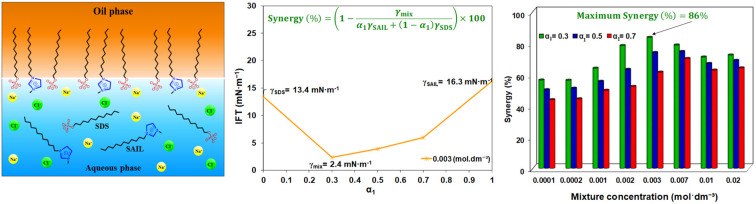

## 1. Introduction

Oil plays an increasingly pivotal role in driving global economic growth. However, a significant portion of crude oil remains inaccessible despite advancements in primary and secondary recovery methods.^1^ Therefore, it is imperative to investigate efficient and practical approaches for chemical enhanced oil recovery (CEOR) utilizing surfactants.^2,3^ Conventional surfactants encounter limitations in application due to their sensitivity to the prevailing salinity and temperature conditions in oil reservoirs.^4^ In contrast, surface-active ionic liquids (SAILs) have emerged as promising candidates owing to their high activity and favorable attributes, including stability, low toxicity, recyclability, and low vapor pressure.^5,6^ Nonetheless, SAIL synthesis is still in its budding stages, and production costs remain relatively high.^7,8^

For effective CEOR, it is crucial to significantly reduce the crude oil–water interfacial tension (IFT) to very low levels. However, the use of SAILs alone may not suffice in achieving this objective. Blending SAILs with conventional surfactants can yield substantial synergy in IFT reduction, offering economically viable and readily available solutions. Studies have shown remarkable improvements in IFT reduction when employing surfactant mixtures in systems such as (toluene + *n*-decane)–water.^9^ Additionally, the synergistic effects of SAILs combined with conventional surfactants have been demonstrated to enhance the interfacial properties of crude oil–water systems, thereby increasing oil recovery efficiencies.^10,11^ Notably, among various types of SAILs, imidazolium-based compounds exhibit superior activity.^12^

Building upon our prior investigations into fundamental CEOR principles,^13,14^ this study focuses on mixtures comprising the long-chain cationic imidazolium-based SAIL, namely 1-dodecyl-3-methylimidazolium chloride, [C_12_mim][Cl], and the anionic conventional surfactant, sodium dodecyl sulfate (SDS). These mixtures offer a potential solution to the cost-intensive nature of SAILs, particularly if synergies can be effectively harnessed. The study aims to evaluate the extent of IFT reduction and critical micelle concentration (CMC) for crude oil–water system. In this regard, considerable IFT and CMC reductions as well as low IFT achievement at CMC are expected alongside high synergy under low total concentration and ambient temperature. In summary, the following objectives are addressed:

• Investigating the individual impacts of the SAIL and SDS components.

• Analyzing the results of individual surfactants using the Gibbs adsorption equation.

• Assessing the degree of synergism in IFT and CMC reductions through mixture utilization.

• Evaluating the results by the Rosen Non-Ideal Interactions in Binary Mixtures (NIBM) theory.

## 2. Experimental

### 2.1. Materials

**Fig. 1:**
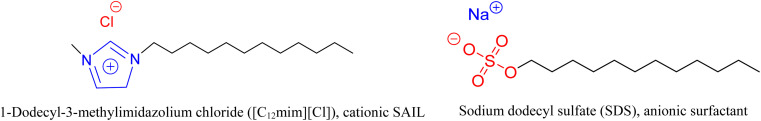
The used SAIL and the conventional surfactant.

To conduct experiments, crude oil was from the Marun oilfield in southern Iran, with the main specifications outlined in Table 1. The synthesized long-chain cationic imidazolium-based SAIL consisted of a twelve-carbon atom alkyl chain and chlorine anions, identified as 1-dodecyl-3-methylimidazolium chloride, and abbreviated as [C_12_mim][Cl]. The synthesis of this product followed as a previously reported method.^15^ The SDS anionic surfactant was a Merck product (99% pure). For clarity, the chemical structures of the imidazolium-based SAIL and SDS surfactant are illustrated in Fig. 1. Aqueous phase solutions were prepared using high-quality, pure distilled water.

**Table 1:** Most important crude oil specifications

Specification/composition	Value
API gravity	20.7
Saturated (wt%)	54.0
Aromatic (wt%)	22.3
Resin (wt%)	6.7
Asphalt (wt%)	7.7
Acidity number (mg KOH per g)	0.09
Sulfur content (wt%)	1.63
Salt content (lbs per 1000 bbls)	4
Water content (wt%)	Nil
Density at 20 °C (g cm^−3^)	0.915
Viscosity at 70 °F (cP)	55
Viscosity at 100 °F (cP)	44
Kinematic viscosity at 70 °F (cSt)	60
Pour point (°F)	10
Flash point (°F)	70
Reid vapor pressure (psi)	12.1
Loss at 200 °C (wt%)	9.3

### 2.2. Instruments and procedures

The IFT (*γ*) was measured using a pendant drop tensiometer (CA-ES10, Fars EOR Technology). Crude oil was dispensed onto the tip of a stainless steel needle which was submerged in the aqueous bulk solution. Detailed descriptions of the experimental setup and methodology can be found in previous publications.^16,17^ IFTs were determined at various intervals by analyzing the geometric characteristics of the pendant drop and processing images with dedicated software.^18^ Utilizing this approach, an equilibrium IFT of 29.1 mN m^−1^ was established for the crude oil-pure water system at 298.2 K. Additionally, the surface tension of water (in contact with air) measured 71.9 mN m^−1^ at the same temperature, closely aligning with the literature-reported value of 72.0 mN m^−1^.^19^ All experiments were conducted under ambient pressure conditions and at a constant temperature of 298.2 K, regulated by a thermostat with an uncertainty of 0.1 K.

Surfactants, both individually and in mixtures, were employed within a concentration range from 1.0 × 10^−4^ to 2.5 × 10^−2^ mol dm^−3^, prepared by mass. The blending of components was guided by the SAIL mole fraction, denoted as *α*_1_ = *C*_1_/*C*_12_ while *C*_1_ represents the molar bulk concentration of the SAIL and *C*_12_ = *C*_1_ + *C*_2_ the total concentration of the SAIL and SDS. The mole fraction (*α*_1_) ranged from 0 to 1, ensuring proper composition. An Anton Paar oscillating densitometer (DMA 4500, Austria) was utilized to ascertain the density of solutions, a crucial factor in determining interfacial tension. The densitometer's uncertainty was at 1.0 × 10^−4^ g cm^−3^. The critical micelle concentration (CMC) was determined as the concentration corresponding to the intersection of tangent lines drawn to the upper and lower regions of the variations in interfacial tension *versus* surfactant concentration.

## 3. Results and discussion

### 3.1. Interfacial tension with individual surfactants

**Fig. 2:**
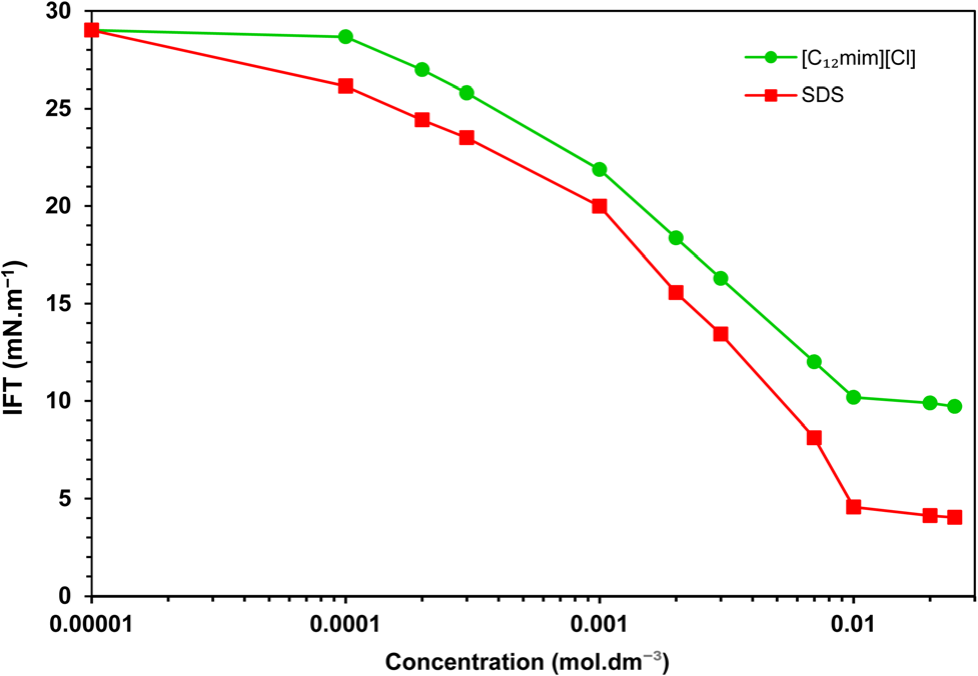
IFT variation in the crude oil–water system *versus* concentration of individual surfactants.

Fig. 2 depicts the IFT variations when individual surfactants were present. A substantial decrease in IFT is evident until surfactant concentrations surpass the CMC. Notably, in the presence of [C_12_mim][Cl] and SDS, the IFT decreases from an initial value of 29.1 to 9.7 and 4.0 mN m^−1^, respectively, with corresponding CMCs detected at 9.8 × 10^−3^ and 9.4 × 10^−3^ mol dm^−3^.

For precise evaluation of individual surfactants, various relevant parameters pertaining to their interfacial behavior were explored. The IFT values at Critical Micelle Concentration (*γ*_CMC_) and the minimum IFT achieved (*γ*_min_) were determined through analysis of the IFT variation with surfactant concentration (Fig. 2). It is noteworthy that the absence of a distinct minimum point in the plot of IFT *versus* concentration near the CMC suggests the purity of the surfactants.^3^ Another pivotal parameter to consider is the effectiveness of IFT reduction, quantified as the interfacial pressure at the CMC (*Π*_CMC_) as:^20^

1 *Π*_CMC_ = *γ*_0_ − *γ*_CMC_

where *γ*_0_ denotes the baseline IFT of the pure system without any additive, indicating the maximum reduction in IFT achievable in the presence of a surfactant. Thus, it serves as a metric for assessing the efficacy of a surfactant.^4^

Moreover, through the application of the Gibbs adsorption equation, it becomes feasible to ascertain the maximum concentrations adsorbed at the interface, denoted as *Γ*_max_, derived from the respective maximum value of the interface concentration:^21^2
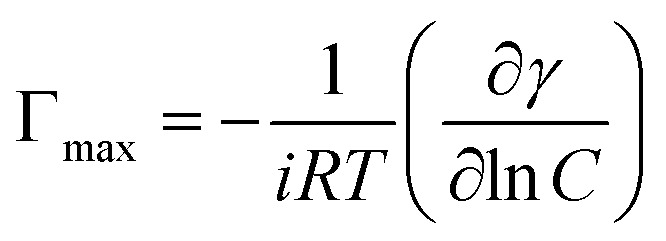
here, *R* and *T* denote the ideal gas constant and the absolute temperature. Hence, the determination of the minimum interface area occupied by each molecule, denoted as *A*_min_, is consequently derived from:3
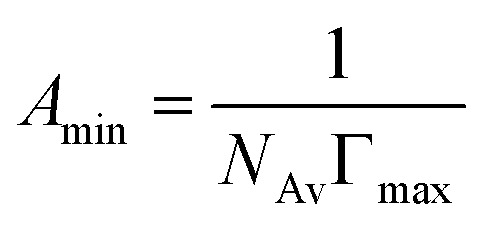
where *C* represents the adsorbate concentration and *N*_Av_, denotes Avogadro's number. The parameter *i* signifies the number of surfactant species. As the surfactants utilized in this study dissociate into a cation and anion, *i* is set to 2.^4^

Moreover, the propensity for micellization can be evaluated through the calculation of the free energy of micellization, represented as Δ*G*_m_ according to:^22^

4 Δ*G*_m_ = *RT* ln(CMC)

alternatively, the standard free energy of adsorption (Δ*G*_ads_) which dictates the spontaneity of surfactant adsorption at the interface may be formulated as:^21^5
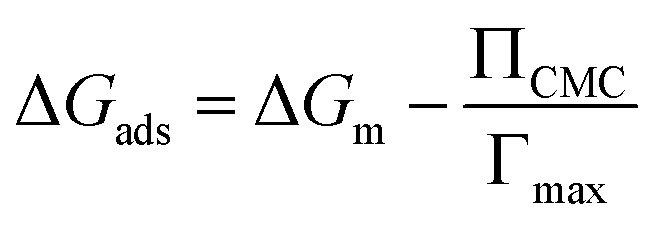


Based on the given equations, the computed parameters for individual surfactant are summarized in Table 2.

**Table 2:** Interfacial parameters corresponding to individual surfactants

Parameter	[C_12_mim][Cl]	SDS
*γ*_min_ (mN m^−1^)	9.7	4.0
Maximum IFT reduction (%)	66.5	86.1
CMC × 10^3^ (mol dm^−3^)	9.8	9.4
*γ*_CMC_ (mN m^−1^)	10.4	4.7
*Π*_CMC_ (mN m^−1^)	18.6	24.3
*Γ*_max_ × 10^5^ (mol m^−2^)	55.1	71.4
*A*_min_ ×10^2^ (nm^2^)	29.2	22.5
Δ*G*_m_ (kJ mol^−1^)	−11.5	−11.6
Δ*G*_ads_ (kJ mol^−1^)	−11.7	−11.8

By examining Table 2 one can easily deduce that SDS exhibits a greater efficiency in reducing IFT compared to the imidazolium SAIL, [C_12_mim][Cl]. SDS demonstrates a remarkable maximum IFT reduction of over 86%, but the imidazolium SAIL achieves a reduction of approximately 67%.

The observed distinction may be linked to the imidazolium SAIL's bulkier structure and increased spatial occupation stemming from the presence of an aromatic ring. This characteristic leads to a lower concentration of adsorbed SAIL molecules at the interface compared to SDS, thus resulting in a less pronounced reduction in IFT. Consequently, the criterion of interfacial pressure, *Π*_CMC_, demonstrates a higher value for SDS, aligning with SDS inherent adsorption tendency. Hence, the elevated value of *Γ*_max_ for SDS, confirms the higher interfacial concentration for it. In parallel, SDS assumes a more condensed orientation at the interface, leading to a reduced interfacial area per adsorbed molecule (*A*_min_) in comparison to the imidazolium-based SAIL.

In Table 2, notably low CMCs are observed for both the components, a consequence of their pronounced hydrophobicity of the long-chain surfactants. Additionally, the almost identical CMC values for SDS and [C_12_mim][Cl] emphasizes their analogous hydrophobic characteristics.

Finally, the negative Gibbs free energy values affirm the mutual tendency of both surfactants to spontaneously adsorb at the interface and form micelles, albeit with a more pronounced tendency for SDS.

### 3.2. Interfacial tension with mixture surfactants

**Fig. 3:**
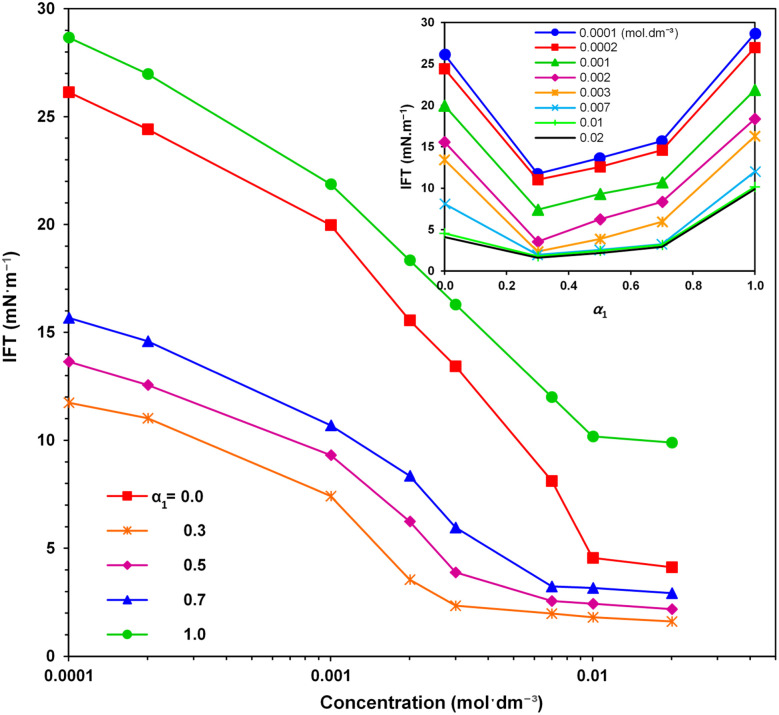
Variation of the crude oil–water IFT *versus* concentration of individual and mixture of surfactants for different mole fractions. Inset figure shows the IFT changes *versus α*_1_ at different mixture concentrations.

Fig. 3 depicts the variations in IFT against mixture concentration for various mole fractions of the SAIL (*α*_1_). It is apparent that, irrespective of the mole fraction, the IFT steadily decreases with increasing surfactant concentration. The ease of adsorption at lower concentrations corresponds to a steeper slope in IFT variation. It is worth noting that low IFT values are desirable as they correspond to high capillary numbers in oil reservoirs.^12,23^

The inset figure further illustrates the alterations in IFT *versus α*_1_ at various mixture concentrations, indicating a notable decrease in IFT with *α*_1_ followed by an ascent toward the corresponding IFT with solely the SAIL. The lowest recorded IFT value of 1.6 mN m^−1^ is associated with the SAIL mole fraction of *α*_1_ = 0.3.

Synergism occurs due to the action of surfactants, SAIL and SDS in this study, where a specific interfacial tension can be achieved at a total mixed surfactant concentration lower than that required for either of surfactants individually.^4^

**Fig. 4:**
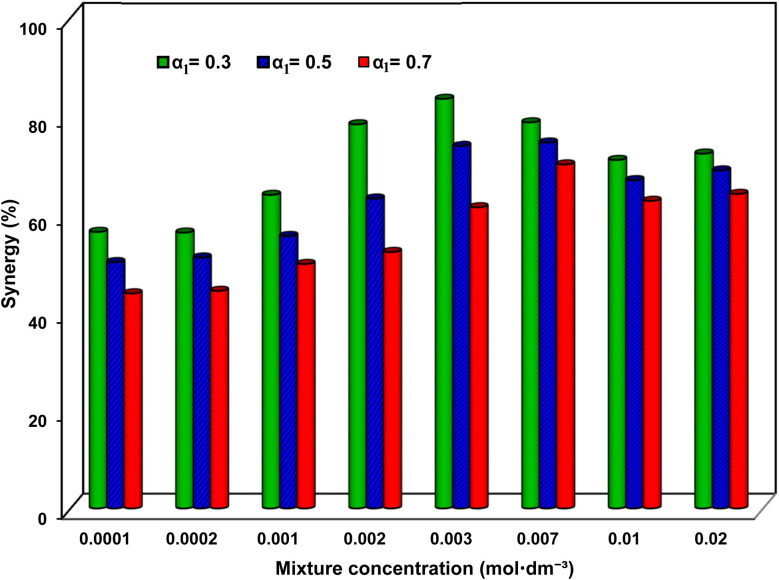
The percentage of synergy in IFT reduction *versus* mixture concentration at different SAIL mole fraction, *α*_1_.

The synergism action of surfactants in IFT reduction could be quantified by comparing the dominant IFT with that obtained from linear contribution of the SAIL and SDS in the mixtures (*i.e.* no synergism) under a specific concentration as:^10,11,24^6
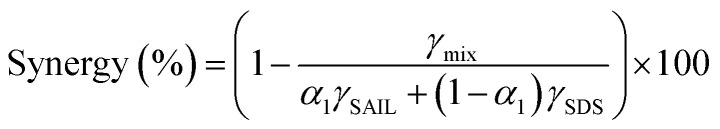
here, *γ*_mix_, *γ*_SAIL_ and *γ*_SDS_ represent, respectively, the system IFT with the mixture, solely with the SAIL, and solely with SDS. Correspondingly, the percentage of synergy in IFT reduction *versus α*_1_ is depicted in Fig. 4 for typical concentrations. Compared to previous investigations on blends of cationic and anionic surfactants^25,26^ as well as SAILs and conventional surfactants,^21,27^ the notably higher degree of synergy, underscores the potent action of the imidazolium-based SAIL and SDS mixtures in this study. Clearly, a significant increase in synergism is achieved at low concentrations, reaching its peak at 0.003 mol dm^−3^, followed by slight decrease up to 0.01 mol dm^−3^, and then remains almost constant. At low concentrations, positively and negatively charged molecules are consistently situated in close proximity, effectively neutralizing electrostatic repulsion and resulting in high synergies. Conversely, at higher concentrations, the dense arrangement of adsorbed molecules leads to minimal change in synergy.

**Fig. 5:**
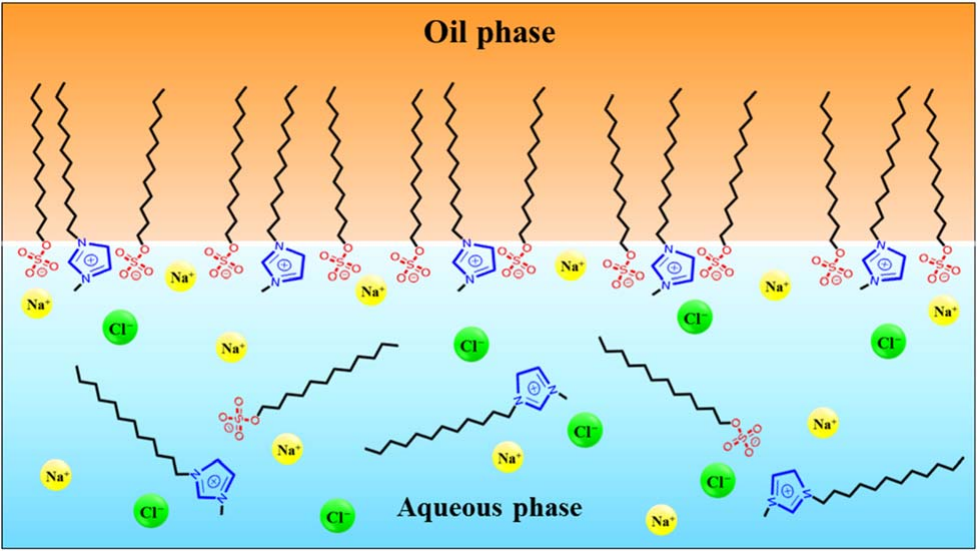
The arrangement of the of the SAIL and SDS molecules at the crude oil–water interface.

As a distinctive SAIL mole fraction, Fig. 4 highlights that the highest degree of synergy (resulting in the lowest IFTs) occurs at *α*_1_ = 0.3, achieving an impressive 83.6% synergy under the mixture concentration of 0.003 mol dm^−3^. Considering the surfactant structures (depicted in Fig. 1), it can be inferred that due to the influence of the SAIL positive charge ring and SDS negative charge, the maximum synergy is potentially associated with the SAIL : SDS molar ratio of 1 : 1. However, the optimal ratio appears to be 1 : 2 (most corresponding to *α*_1_ = 0.3). This finding holds significance as it indicates that an optimal mixture with a low SAIL contribution is highly efficient. This can be attributed to the bulky SAIL head group and the charge distribution in the aromatic ring, facilitating the attraction of two SDS molecules alongside each SAIL.^28^Fig. 5 illustrates the most probable arrangement of the surfactant molecules at the oil–water interface.

**Fig. 6:**
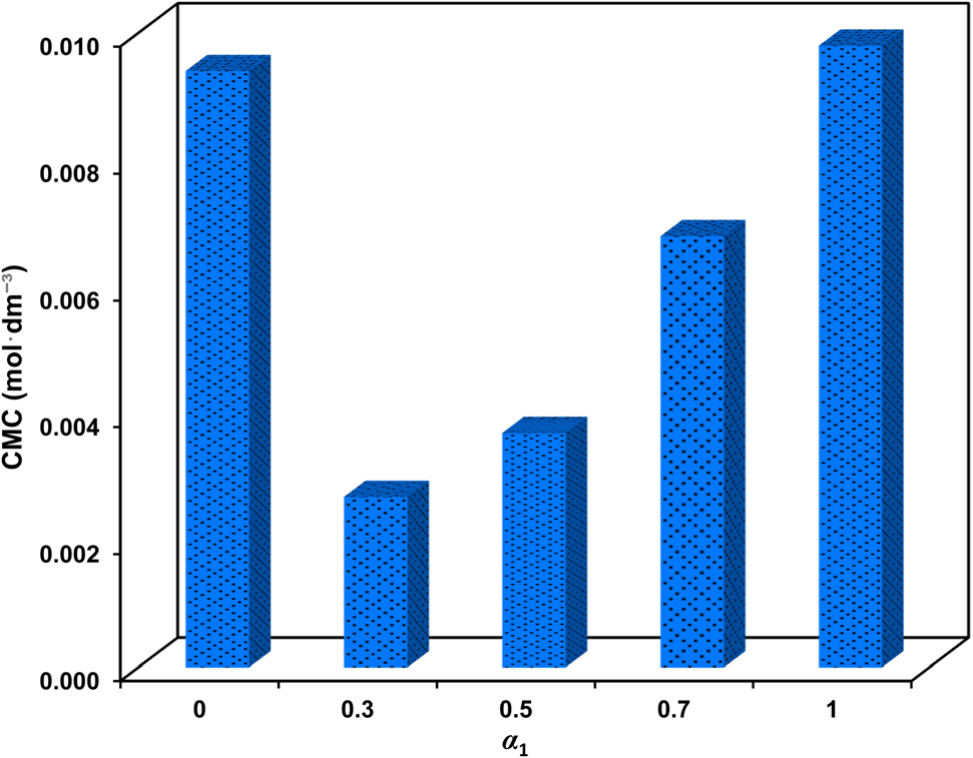
The CMC values for different mole fractions of surfactants mixture.

Fig. 6 illustrates the variation of CMC of the mixtures concerning the SAIL mole fraction. It is noteworthy that CMC decreases to an exceptionally low value of 2.7 × 10^−3^ mol dm^−3^, corresponding to a 72.1% CMC synergy (in comparison to the linear contribution of surfactants in the mixture) at *α*_1_ = 0.3. Consistent with the aforementioned findings, the intermolecular attractive forces between the surfactants weaken the electrostatic repulsion; thus facilitating the formation of micelles at lower concentrations. It is imperative to highlight the significance of a low CMC in CEOR processes due to the transportation of oil droplets *via* surfactant flooding.^29,30^

Table 3 provides comparisons between the interfacial parameters using mixtures of SAIL/surfactant in water–crude oil system. Compared to other investigations,^26,31–34^ outstand findings are confirmed within the present study. By using only total concentration of 0.003 mol L^−1^, a high reduction in the IFT and *γ*_CMC_, compared to the sole SAIL, was obtained with low ionic liquid mole fraction of 0.3 at room temperature of 25 °C leading to synergy of 83.6%. Jia and co-workers^32^ used the same SAIL at higher temperature with a crude oil from Karamay oil field (China). Though much higher total concentration, *C*_12_, no significant reduction in *γ*_CMC_ was achieved compared to the present study.

**Table 3:** Comparing results with other related studies

Crude oil source and type	Used IL	*T* (°C)	*α*_1_	*C*_12_ (mol L^−1^)	Max. *γ* reduction (%) compared to sole SAIL	*γ*_CMC_ reduction (%) compared to sole SAIL	Reference
Shengli (China), heavy	1-Dodecyl-3-methylimidazolium bromide	30	0.33	0.002	99	—	26
Ankleshwar (India), light	1-Hexadecyl-3-methyl imidazolium bromide	35	0.80	0.220	20	50	31
Karamay (China), heavy	1-Dodecyl-3-methylimidazolium chloride	30	0.33	0.017	86	27	32
Tapis (China), light	Choline laurate	25	0.40	0.219	72	—	33
Arab (Saudi Arabia), light	1-Butyl-3-methylimidazolium lauroyl sarcosinate	25	0.83	0.177	46	—	34
Marun (Iran), heavy	1-Dodecyl-3-methylimidazolium chloride	25	0.30	0.003	86	77	Present work

The Non-Ideal Interactions in Binary Mixtures (NIBM) theory was utilized to analyze the obtained results and determine the adsorbed SAIL mole fraction (*X*_1_) as well as the molecular interaction parameter (*β*) according to the following equations:^4^7
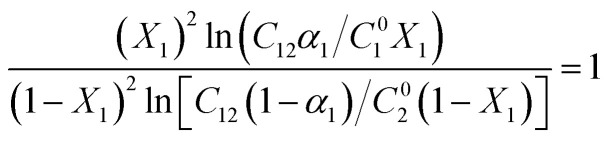
8
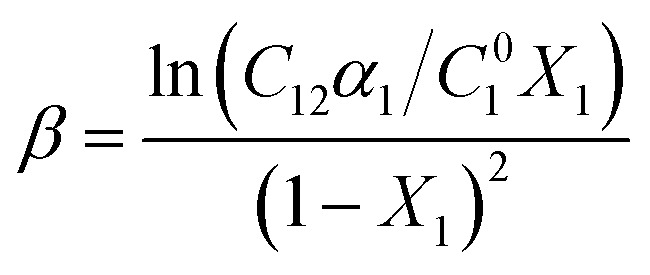
where *C*^0^_1_, *C*^0^_2_ and *C*_12_ denote the bulk concentration of the SAIL, SDS, and their mixture, respectively, all corresponding to a certain IFT. These concentrations were derived from IFT variations *versus* individual surfactant concentration and their mixture for a particular *α*_1_ value (see Fig. 3). Consequently, accurate values of *X*_1_ and *β* were calculated from eqn (7) and (8) using an iteration method.^35^ Negative *β* values validate an attractive molecular interaction, while positive values represent repulsion. The interfacial tension reduction efficiency by a surfactant has been explained as the surfactant concentration in solution phase required to yield a given interfacial tension.

What's more, considering the principles of the NIBM, the prerequisites for synergistic effects to manifest in the enhancement of interfacial tension reduction efficiency are as follows:^4^

• *β* must be negative.

• The absolute value of |*β*| should be more than |ln(*C*^0^_2_/*C*^0^_1_)|

These conditions were satisfactory for the interfacial tension data in this work. It has been recommended to establish the value of *β* based on *C*^0^_1_, *C*^0^_2_ and *C*_12_ values derived from the IFT–log *C* plots, ensuring that the slopes remain predominantly linear. In pursuit of this, one may extend a plot beyond the CMC of SAIL, extrapolating linearly from the section exhibiting maximum slope just before reaching the CMC.^4^

**Fig. 7:**
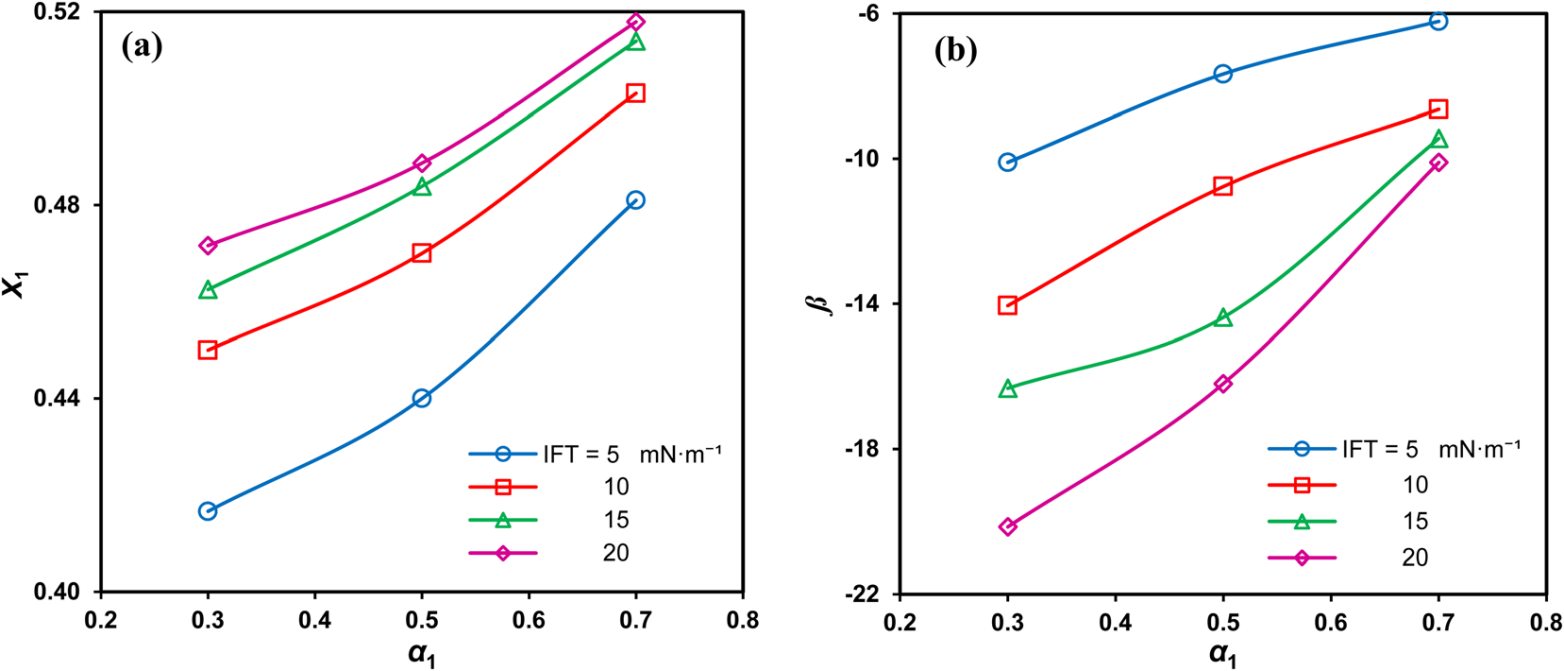
Interface mole fraction (a) and interaction parameter (b) *versus* the SAIL mole fraction for various IFTs.

Fig. 7(a) demonstrates that the adsorbed SAIL mole fraction (*X*_1_) increases with a rise in its mole fraction. However, this increase in *X*_1_ is less pronounced than expected, despite the greater mole fraction suggesting increased adsorption and heightened interfacial activity of SDS. Moreover, as the interface concentration increases and the IFT decreases, *X*_1_ diminishes, indicating a stronger affinity of SDS for interfacial absorption compared to the imidazolium-based SAIL.

The negative *β* values, as shown in Fig. 7(b), indicate that despite self-repulsions among individual surfactant molecules, there is a prevailing attractive interaction between the adsorbed components in mixtures. Moreover, high absolute *β* values consistently signify a strong synergistic effect.^36^ The highest absolute interaction is observed at *α*_1_ = 0.3, aligning with previous findings. Additionally, as the IFT decreases, the absolute *β* values decrease due to higher interfacial concentrations and the closer arrangement of the adsorbed surfactant molecules. This, in turn, intensifies the repulsion between similarly charged molecules.

## 4. Conclusions

The study explored the effects of blends of a long-chain cationic imidazolium-based SAIL, [C_12_mim][Cl], and the anionic conventional surfactant, SDS, on the interfacial tension and critical micelle concentration within the crude oil–water system.

Initial experiments validated the effectiveness of individual surfactants in reducing IFT. Specifically, SDS demonstrated the most substantial IFT reduction highlighting the superior effectiveness of SDS. Through assessment was also made on the effectiveness of each individual surfactant and a range of theoretical parameters linked to their interfacial behavior were analyzed.

The adaptive charge interactions within the surfactant blends facilitated a significant reduction in the IFT, surpassing the effectiveness achievable by individual components alone. The blends exhibited optimal performance at a SAIL mole fraction of only 0.3, resulting in remarkably low IFTs. These variations in IFT aligned well with the predictions of the NIBM model, and the theoretical parameters showed reasonable consistency. Additionally, the CMC decreased to a minimum value in the presence of the mixtures.

All in all, at ambient temperature and low total concentration, high reductions in IFT, CMC and IFT at CMC alongside high synergy could highly diminish the operating cost by employing mixtures of SAILs and conventional surfactants in enhanced oil recovery. However, to fully capitalize on their extensive application potential, further research is warranted, particularly under conditions of high salinity and varying pressure/temperature environments.

## Author contributions

The manuscript was written through the contributions of all the authors. All authors have given approval to the final version of the manuscript.

## Conflicts of interest

There are no conflicts of interest to declare.

## Supplementary Material
